# Management of Hepple Stage V Osteochondral Lesion of the Talus with a Platelet-Rich Plasma Scaffold

**DOI:** 10.1155/2017/6525373

**Published:** 2017-03-16

**Authors:** Wenqi Gu, Tanzhu Li, Zhongmin Shi, Guohua Mei, Jianfeng Xue, Jian Zou, Xiaokang Wang, Haotong Zhang, Hongwei Xu

**Affiliations:** ^1^Shanghai Jiaotong University Affiliated Sixth People's Hospital, Shanghai 200233, China; ^2^The People's Hospital of Shigatse City, Shigatse, Tibet 857000, China

## Abstract

There has been no consensus on the treatment or prognosis of Hepple stage V osteochondral lesions of the talus (OLTs), especially for lesions greater than 1.5 cm^2^ in size. The objective of this study was to investigate the clinical outcomes achieved upon application of a platelet-rich plasma (PRP) scaffold with a cancellous bone autograft for Hepple stage V OLTs. Fourteen patients (mean age, 39 years) were treated with a cancellous bone graft and a PRP scaffold between 2013 and 2015. The mean time to surgical treatment was 23.5 months. Ankle X-ray and magnetic resonance imaging were performed at the final follow-up. Functional outcomes were evaluated according to the Visual Analog Scale (VAS) score, American Orthopaedic Foot and Ankle Society (AOFAS) score, and Short Form 36 (SF-36) score. The range of motion (ROM) of the ankle joint and complications also were recorded. Thirteen patients completed the full follow-up, with a mean follow-up duration of 18 months. MRI demonstrated the complete regeneration of subchondral bone and cartilage in all patients. The postoperative VAS, AOFAS ankle and hindfoot, and SF-36 scores were improved significantly (all *P* < 0.001) without obvious complications. We suggest that, for the Hepple stage V OLTs, management with cancellous bone graft and PRP scaffold may be a safe and effective treatment.

## 1. Introduction

Osteochondral lesions of the talus (OLTs) often cause pain and disability and present a great challenge to foot and ankle surgeons. The size of lesions varies, and a subchondral cyst can develop if the lesion is left untreated. In 1999, Hepple and colleagues developed a new classification system for OLTs with five stages according to MRI manifestation [1]. The identifying characteristic of a stage V lesion is the formation of subchondral cyst, which makes treatment more difficult. The key point of management for this type of lesion is to reconstruct and repair the subchondral bone and cartilage simultaneously. Although various procedures for treating subchondral cystic OLTs have been reported, including arthroscopic microfracture, retrograde arthroscopic procedure, autograft or allograft of osteochondral peg, and chondrocyte transplantation [2–6], the best treatment approach remains controversial. The arthroscopic microfracture technique is widely preferred by surgeons due to the advantages of minimal invasion and supporting quick recovery, but the success rate has only been satisfactory for cases of small- or mid-sized lesions [2]. Large lesions (>1.5 cm^2^) with subchondral cyst tend to respond poorly to microfracture. The retrograde arthroscopic technique is ideal for large cystic lesions with intact cartilage [5]. However, in most cases, the cartilage over the lesion site is also unstable and prone to dehiscence. The osteochondral peg autograft or allograft may be a reasonable option for these lesions, because it allows simultaneous restoration of cartilage and subchondral bone. However, the undesirable donor site morbidity [7], curvature mismatch, and healing rate remain problems [8]. Chondrocyte transplantation may be a promising approach, but the high expense and two-stage operation are major challenges in China. Based on the disadvantages of the above-mentioned techniques for application in cases of Hepple stage V OLTs, we have attempted to develop a reliable technique for repairing this type of lesion.

Cancellous bone autograft is a reasonable technique for repairing a subchondral bone defect after debridement of a cyst that avoids obvious donor site injuries. However, the repair of cartilage lesion remains a great concern. Platelet-rich plasma (PRP) has been proposed as a novel treatment modality for the management of articular cartilage injuries, wound healing, nonunion, muscle injury, and tendon disease [9–13]. The potential of PRP for cartilage repair has been demonstrated through its anabolic effect on chondrocytes, mesenchymal stem cells (MSCs), and synoviocytes as well as its ability as a bioactive cell scaffold to fill defects and enhance cartilage regeneration [14]. Animal experiments also demonstrated that adjunctive use of PRP produced a better healing response than a mosaicplasty-only procedure for osteochondral lesions [15]. Currently, PRP is mainly applied as an additional postoperative therapy [16]. Considering the great potential of PRP for cartilage repair and regeneration, we applied a cancellous bone autograft with a PRP scaffold for treatment of Hepple stage V OLTs.

The objective of this study was to investigate surgical techniques and clinical outcomes of using a PRP scaffold with a cancellous bone autograft to repair a Hepple stage V OLT. We hypothesized that PRP would be a reliable scaffold for cartilage regeneration and be beneficial to the healing process for a large Hepple stage V OLT.

## 2. Material and Methods

### 2.1. Patient Recruitment

The current study was approved by Shanghai Sixth People's Hospital ethics review board. All participants signed an informed consent form before the study.

The inclusion criteria of this study were (1) stage V OLT according to MRI classification by Hepple et al. [1]; (2) lesion size > 1.5 cm^2^; (3) age from 18–60 years; (4) primary or revision procedure; (5) normal ankle and hindfoot alignment; and (6) involvement of medial dome with cartilage dehiscence. The exclusion criteria were (1) diffusive degenerative joint changes; (2) inflammatory arthritis or chronic inflammatory disease; (3) history of infection; (4) malalignment of ankle and hindfoot; (5) nonreconstructable defect; (6) body mass index > 30 kg/m^2^; (7) central or lateral lesion; and (8) subtalar joint involvement. Complete clinical and radiographic evaluations were carried out preoperatively, including X-ray examination, computed tomography (CT) scanning, and MRI scanning of the ankle joint. The preoperative Visual Analog Scale (VAS) score, American Orthopaedic Foot and Ankle Society (AOFAS) ankle and hindfoot score, Short Form 36 (SF-36) score, and range of motion (ROM) of the ankle joint were also documented.

### 2.2. PRP Scaffold Preparation

PRP scaffolds were prepared using the WEGO Platelet-Rich Plasma Preparation Kit (WEGO Ltd., Shandong, China). Approximately 40 mL of blood was taken from patient's arm, placed in a tube provided in the kit, and spun twice in a portable centrifuge (WEGO Ltd.) at 2000 rpm for 10 minutes each time. This standard process produced 3-4 mL of plasma.

### 2.3. Surgical Technique

**Figure 1:**
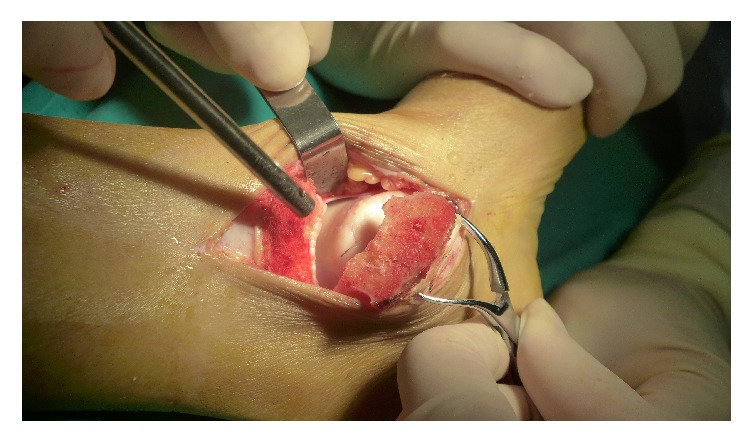
A medial approach followed with a medial malleolar osteotomy was performed.

Following the induction of general anesthesia, the patient was laid supine on the operating table with a pneumatic tourniquet applied to the thigh. The iliac crest region was prepared. For patients with an anteromedial lesion, a standard anteromedial approach was made from the anterior border of medial malleolar, which lay between the anterior tibial tendon and posterior tibial tendon, to the navicular tubercle. After dissection of the superficial layer of the deltoid ligament and plantar flexion of the ankle joint, the anteromedial lesion was visualized. For patients with a centromedial or posteromedial lesion, a medial approach was performed from the center of medial malleolar to 6-7 cm proximally and to the navicular tubercle distally. A capsulotomy was made after dissection from the anterior medial malleolar. After positioning the osteotomy line by a guide K-wire, the medial malleolar osteotomy was performed using an oscillating saw with an osteotome for the final cut. The medial malleolar fragment was rotated for exposure, and the lesion area was confirmed (Figure 1).

**Figure 2:**
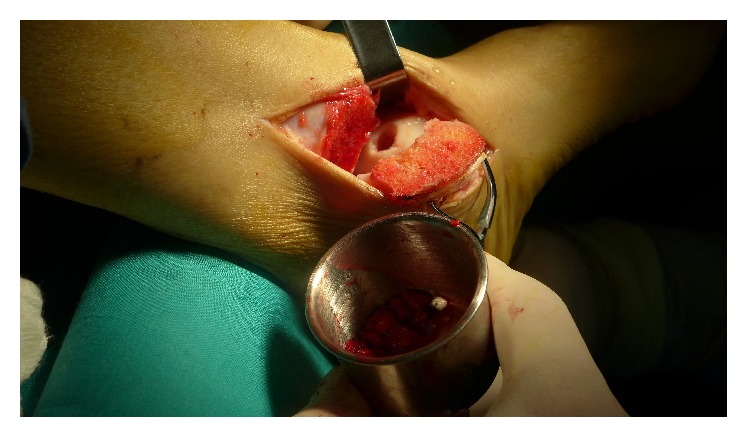
After elevation of injured cartilage, the cyst was debrided and prepared for cancellous bone autograft.

**Figure 3:**
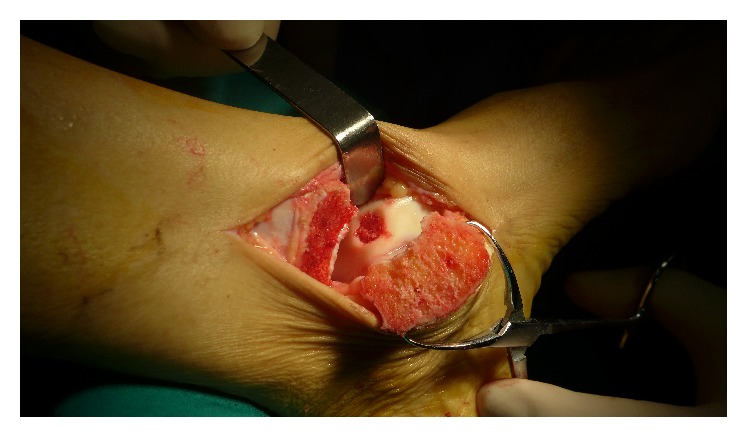
The subchondral defect was filled with cancellous bone.

**Figure 4:**
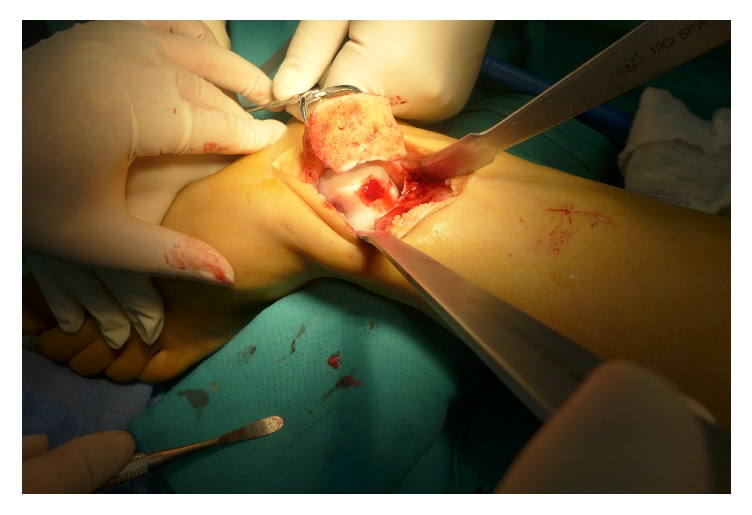
The PRP scaffold coverage was performed.

After elevation of unstable cartilage, the sclerotic subchondral lamella was resected with a mini osteotome, and the cyst was debrided with a curette (Figure 2). After the defect size was measured, a 2.0 mm K-wire was used for drilling of the sclerotic subchondral bone. Next, the cancellous bone autograft was harvested from the iliac crest. The autograft was designed to fill the defect to about 2 mm below the cartilage (Figure 3). The superficial layer of the cancellous bone was covered by the prepared PRP scaffold (Figure 4).

For patients in whom medial malleolar osteotomy was performed, reduction of the medial malleolar fragment was achieved before fixation with two 4.3 mm cannulated screws (Qwix, Newdeal, France). After fluoroscopic evaluation, the capsule and wound were sutured. For the patients with lateral instability, lateral collateral ligament reconstruction was performed via a lateral approach.

### 2.4. Postoperative Management

The RICE (rest, ice, compression, and elevation) principle was applied with a short-leg cast for immobilization. Toe, knee, and hip ROM exercises were started on the second day postoperatively. All patients had postoperative follow-up assessments at 2 weeks and 1, 2, 3, 6, 12, and 24 months after the operation. The wound was checked at 2 weeks postoperatively. If no soft tissue problem was observed, the skin sutures were removed. Patients were fitted with a walking boot after 2 weeks and began ankle ROM exercises. Weight-bearing was permitted at 3 months postoperatively still with the protection of the walking boot. The anteroposterior and lateral view on plain X-ray and MRI scanning were taken during the follow-up. The clinical outcome was evaluated by the VAS, AOFAS ankle and hindfoot, and SF-36 scores. The ROM of the ankle joint was also measured. The neutral position of the ankle was defined as 0°. All complications were recorded during the follow-up assessment.

### 2.5. Statistical Analysis

SAS 8.0 (SAS Institute Inc., Cary, NC, USA) was used for statistical analysis. The *t*-test was applied for comparison of preoperative and postoperative ROM of the ankle joint, VAS score, AOFAS score, and SF-36 score. *P* < 0.05 represented a statistically significant difference.

## 3. Results

Fourteen patients who were treated between 2013 and 2015 in our hospital were enrolled in this study. The study population included 10 males and 4 females with an average age of 39 years (range, 21–57 years). The mean time from onset to surgical treatment was 23.5 months (range, 11–48 months). Nine cases (64.3%) were left side involved, and five cases (35.7%) were right side involved. Of the 14 patients, 10 (71.4%) patients had a history of ankle sprain, and 4 (28.6%) patients had spent a long period for professional sports. Three (21.4%) patients underwent a primary arthroscopic procedure, whereas the others had received conservative therapy for at least 6 months. Lateral instability was seen in three (21.4%) cases. The average lesion size was 2.1 cm^2^ (range, 1.6–3.0 cm^2^). According to the measurements on MRI, 8 patients were 1.6–2.0 cm^2^, 4 cases were 2.1–2.5 cm^2^, and another 2 cases were 2.6–3.0 cm^2^. According to Raikin and Elias's talar dome 9-zone grid system [17], 8 cases had a lesion in zone 4 (centromedial zone), 5 cases had a lesion in zone 7 (posteromedial zone), and 1 case had a lesion in zone 1 (anteromedial zone).

**Figure 5:**
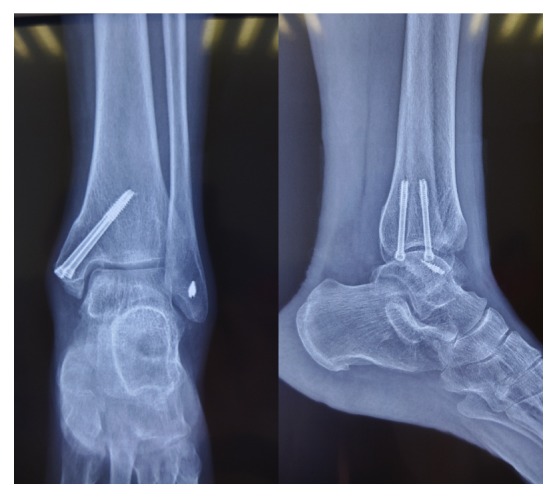
The series of X-ray demonstrated the bony union of osteotomy site on the 3rd month postoperatively. The collateral ligament was also repaired in this patient.

**Figure 6:**
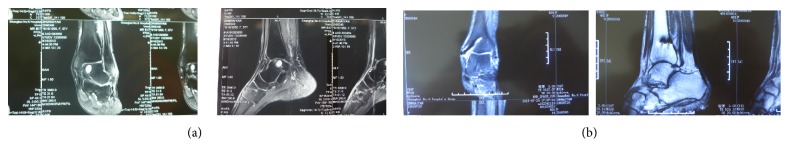
The MRI manifested that the large size of subchondral cystic lesion (a) was successfully repaired with a congruent curvature (b).

All the wounds healed without complications of infection or skin necrosis. Thirteen patients completed the full follow-up for an average of 18 months (range, 12–24 months). One patient was lost to follow-up after suture removal. Postoperative plain X-ray demonstrated that bony union of the osteotomy site was achieved in all patients within 3 months without malunion (Figure 5). The VAS score, AOFAS ankle and hindfoot score, SF-36 score, and ROM of ankle joint were all improved significantly postoperatively (Table 1). MRI manifested the restoration of the subchondral bone and a good congruence and curvature of the regenerated cartilage along the surrounding cartilage in all cases (Figure 6). No cases of implant failure, recurrence of lesion, or degenerative arthritis occurred during the follow-up, and no patients required a second stage of operation.

## 4. Discussion

Hepple stage V OLTs with a subchondral bone cyst are great challenges for foot and ankle surgeons. Most lesions of this stage are symptomatic and require surgical management, but limited data are available regarding the best surgical strategy and corresponding clinical outcomes. The key point for this type of lesion is to simultaneously address the subchondral defect and cartilage lesion. This study aimed to investigate a new method for treating this type of lesion.

For the management of OLTs, the selection of the treatment protocol depends on various factors, including patient age, lesion size, and cyst formation, which all play important roles in the prediction of prognosis. Some patients with OLTs may benefit from a primary arthroscopic surgery regardless of the existence of a subchondral cyst [2]. However, the size of the lesion is a major predictive factor of prognosis after the arthroscopic procedure, which may be less suitable for large-sized lesions greater than 1.5 cm^2^ [2, 18]. Robinson et al. reported an unsatisfied outcome in 53% patients of cystic lesion [19]. In the present case series, all of the patients had a large lesion with an average area of 2.1 cm^2^; however we achieved a satisfied outcome. The retrograde technique may be ideal for large OLTs with a subchondral cyst and intact cartilage [5]. The use of some cannulated systems may simplify the procedure and facilitate accurate bone graft. However, in our patient group, cartilage dehiscence was confirmed in all patients preoperatively, which counter-indicated the use of the retrograde technique. Jeong et al. [20] reported the retrograde technique for subchondral cystic OLTs. A progressing arthritic change of ankle joint was confirmed at the first year postoperatively. And they concluded that retrograde procedure might not be theoretically correct and would damage the uninjured bone marrow. The osteochondral autograft may be an acceptable technique for treating stage V OLTs based on the simultaneous restoration of the subchondral bone and cartilage. However, the donor site morbidity is a great concern in the knee-to-ankle procedure, which was reported as high as 16.9% [21], and may cause pain for a period of time [22]. Moreover, for a large size of cystic OLT, a larger subchondral bone peg is needed for autograft, which may increase the risk of donor site injury. However, with a cancellous autograft technique, the donor site symptoms did not occur in our study.

Cancellous bone autograft is an appropriate method with limited injury to the donor site that offers better restoration of the mechanical properties of the talus. A fresh cancellous bone graft populated with native cells was used to facilitate the regeneration and growth of subchondral bone. In our patients, from the follow-up X-ray and MRI we observed a satisfied regeneration of the subchondral bone without bone resorption or recurrence of the cyst, indicating that cancellous bone autograft is an effective method for treating subchondral cysts. Furthermore, the successful reconstruction of the subchondral bone significantly relieved the patients' symptoms, as the postoperative VAS score was obviously improved and no donor site morbidity was seen in this study.

Repair of cartilage lesions is another challenge. After elevation and debridement of injured cartilage, the resulting defect will be hardly healed due to the limited ability of cartilage regeneration. PRP has been proven to have protective effects against chondrocyte apoptosis, inhibit inflammatory processes, improve the cartilage repair, and stimulate the migration and chondrogenic differentiation of human subchondral progenitor cells [23–27]. Thus, application of PRP offers a new method for cartilage repair [9, 28]. In spite of these promising features, PRP has been mainly applied via injection as an additional method for treating OLTs in previous research [16, 29], and the effects of PRP on the repair of large cartilage lesion have been rarely reported. Our application of a PRP scaffold after cancellous bone autograft has several advantages. First, it is convenient and safe to prepare the PRP without obvious donor site morbidity and complications. In this study, no complications associated with PRP collection were observed. In addition, as described above, curvature mismatch is a difficult problem associated with a subchondral peg autograft that sometimes causes symptoms and compromises the clinical outcome. However, with the PRP scaffold, we could repair and restore the cartilage with congruence along the curvature. Furthermore, the patients in this group were young, implying a better chondrocyte viability, greater regeneration capacity, and less degeneration of cartilage. Therefore, for young patients, cartilage repair is more likely to be achieved with the support of a PRP scaffold. During the follow-up, we applied MRI not only for the prediction of prognosis, but also to confirm the restoration of the subchondral bone and cartilage and the congruity of the facet, which is a positive and scientific method for assessment. The follow-up MRI demonstrated that the regenerated cartilage had a good shape and thickness, matching that of the surrounding cartilage, which suggested a better match of properties with native cartilage. Better congruity of the facet is expected to benefit symptom relief and functional recovery. Therefore, we achieved a significant functional improvement and relief of pain in our study.

This study still has some limitations. First, the sample size of this study was small and the control group was not set up for comparison in this study. However, Hepple stage V lesion is uncommon in the clinical work, which may make a comparative study more difficult. Then, the follow-up time was not long enough and long-term outcome remains unknown. Third, the type and characteristics of the regenerated cartilage were unknown and should be confirmed by repeated arthroscopy with histological evaluation, which is less practicable in China. These limitations will be attempted to address in the future. Last, this study excluded patients with a central or lateral lesion. As acceptable clinical outcomes were obtained in this preliminary study, we may expand the application of this approach.

## 5. Conclusion

Application of a PRP scaffold with a cancellous bone autograft in the treatment of Hepple stage V OLTs may achieve acceptable short-term clinical outcomes with better regeneration of cartilage and subchondral bone with better congruity and minimal complications. Closer observation of the effects of PRP on Hepple stage V OLTs is recommended.

## Figures and Tables

**Table 1:** The comparison of clinical evaluation.

	VAS (mean ± SD)	AOFAS (mean ± SD)	SF-36 (mean ± SD)	Plantar flexion (mean ± SD)	Dorsiflexion (mean ± SD)
Pre-op	6.1 ± 1.5 (4*～*9)	54.0 ± 10.6 (31*～*73)	62.0 ± 5.9 (50*～*70)	22.8 ± 8.9° (5°*～*36°)	8.0 ± 3.4° (0°*～*12°)
Post-op	1.1 ± 1.0 (0*～*3)	86.2 ± 6.4 (74*～*100)	85.3 ± 5.9 (73*～*95)	35.5 ± 6.0° (25°*～*45°)	17.2 ± 3.1° (12°*～*22°)
*P* value	*P* < 0.0001	*P* < 0.0001	*P* < 0.0001	*P* < 0.0001	*P* < 0.0001
